# Evaluation of the development process and effects of a foot care program with educational tools for nurses and care workers as in-home service providers

**DOI:** 10.1186/s13104-020-05263-3

**Published:** 2020-09-05

**Authors:** Kashiko Fujii, Minna Stolt

**Affiliations:** 1 Graduate School of Medicine, School of Health Sciences, Nagoya University, 1-1-20 Daiko-Minami, Higasi-ku, Nagoya, Aichi Japan; 2 Department of Nursing Science, University of Turku, Turku, Finland; 3 Turku University Hospital, Turku, Finland

**Keywords:** In-home service providers, Foot care education, Nurses and care workers

## Abstract

**Objective:**

Nurses and care workers who provide in-home services play important roles in assessing and providing care for older people who lack foot self-care abilities. We aimed to evaluate the development process and effects of a foot care program with educational tools for nurses and care workers as in-home service providers. This is a process evaluation with a descriptive mixed-methods study of quantitative and qualitative data conducted from July to October 2019 in Japan.

**Results:**

Foot care education tools were developed to address the issues faced by participants with various work patterns and insufficient foot care education in Japan. The contents of these tools were discussed by a panel and reviewed by experts. Three outcomes were analyzed using descriptive statistics and Pearson’s correlation. Changes in foot care practice scores were significantly correlated with performance scores. The evaluations of five of the eight field nurses suggested that excess information was included in the foot care booklet. Overall, 29 nurses and care workers showed higher than average evaluation scores [3.8–4.1 (standard deviation, 0.62–0.91)] for the motion pictures and PowerPoint presentation. A program according to this conceptual framework must be established and periodically evaluated for refinement.

*Trial Registration* The trial registration number for the University Hospital Medical Information Network is UMIN000036307. Registration Date—2019/07/25

## Introduction

Nurses and care workers (NCWs) working in-home settings or communities face serious challenges because of the markedly increasing aging population worldwide [1]. Demographic changes are a serious national issue in Japan. Therefore, the Japanese government has urgently called for a community-based integrated system to allow older people to live the rest of their lives in their own ways in the familiar environments by using this system [2].

Community-dwelling older people in Japan experience various health conditions, with some requiring assistance for medical treatment or activities of daily living and some using long-term care insurance [3]. Older people may request foot care from NCWs or may refuse to seek active care [4] despite the high prevalence of foot problems in this population. However, studies on this topic are limited [3, 5].

In older people, an inability to bend to cut the nails, impaired vision and fine hand movements [6, 7] results in the lack of foot self-care, possibly leading to foot problems [8, 9]. Nurses and care workers may often overlook foot care or related issues due to time constraints at work and limited perception of the significance of the feet. Currently, the limited number of reports on foot care indicates researchers’ lack of interest in this topic.

Nurses and care workers are the key people responsible for identifying foot problems because care activities often require physical contact. In Japan, given the lack of foot care specialists, NCWs require more autonomy to assess and care for foot problems. Therefore, a foot care program was developed by the first author with various educational tools to comply with NCWs’ diverse work patterns and foot care knowledge and practices. We evaluated the development process and effects of a foot care program with educational tools for NCWs. The research questions were as follows: (1) Was the evaluation process of the educational tool development effective? (2) Is there an association between the performance scores obtained using educational tools and improvement in foot care?

## Main text

### Methods

#### Study design

This is a process evaluation with a descriptive mixed-methods study of quantitative and qualitative data conducted from July to October 2019 in Japan.

#### Conceptual framework

Educational tools for the foot care program comprised materials and kits. Tools were used in training sessions and were created based on a conceptual framework generated using all collected resources.

This study was not limited to the item pools of other studies on foot care [10, 11]. It included the effects of prolonged sedentary or toe movements [12–15] and differentiation between soft tissue and lymphatic massages based on their physical implications [16, 17]. Educational tools were created using five steps: full planning, draft creation, evaluation initiation (before implementation), process evaluation (immediately after implementation), and impact evaluation (after intervention).

#### Tool creation

Most of the ideas for the tools in the present study were based on previously reported interventions for diabetic patients. A Power Point presentation (PPT), pamphlets, foot care kits, and hands-on skill sessions were used [18–22]. Interventions using telemedicine or mobile phone text messages and telephone follow-ups were also used as references to obtain ideas, as previously reported [22–26].

To create the tools, an illustrator created original drawings appropriate for the content. The animation characteristics were developed for this study with regard to originality and familiarity. The effects of illustrations have been investigated [27]. Reviews of previous studies using video lectures [28–31] and picture story cards [32] as well as development guidelines [33] were used as references to create the program. All practice programs were based on laws and regulations of Japan and the interpretation reports of the related article by the Ministry of Health, Labor and Welfare, which determine foot care practices that can be performed based on qualifications.

#### Program evaluators

Overall, 3 types of evaluators were included in this study:

1. A total of 36 NCWs working as “in-home service providers,” i.e., those providing home-visit services, 1-day service, or day care service [34], who participated in a 2-month intervention study of foot care programs [35]. This study focused on the evaluation of the development process and overall effects of the program. Therefore, 36 of the 54 initial participants in the intervention group answered all performance questions at post-intervention and 80% or more of the knowledge and practice questions at pre- and post-intervention were considered as evaluators.
2. Eight field nurses who attended a monthly pressure ulcer study group in the T area (similar to a province) in Japan.
3. A total of 29 randomly selected participants from the intervention group [35]. after the first intervention session.

#### Procedure of program evaluation

Before the foot care intervention started, eight nurses evaluated foot care booklet and motion pictures (MPs) using an evaluation sheet for convenience and comprehension. The first author (KF) visited 11 in-home service providers 3–5 times for the intervention groups. After the first intervention session, randomly selected participants from the intervention group evaluated the MPs and PPT. Participants had experienced other sessions, including training and follow-up sessions. Foot care booklet, picture flip cards, and foot assessment sheets were given to the provider, and a one-point advice card was provided to each participant. During the post-intervention, the participants were asked to provide answers regarding the performance of the programs. At both the pre- and post-intervention, they were asked to provide answers regarding foot care knowledge and practice questionnaires. Performance scores and changes in the knowledge and practice scores of NCWs as program participants were calculated.

#### Outcomes

The primary outcome was the association between changes in foot care knowledge and practice scores and performance scores obtained using questionnaires and an evaluation sheet. The secondary outcome was the evaluation by eight field nurses before the intervention. The tertiary outcome was a process evaluation by the 29 participants after the first intervention session.

#### Instruments

Five types of evaluation sheets (an evaluation sheet for MPs and foot care booklet, an evaluation sheet for the PPT and MPs, foot care knowledge and practice questionnaires, performance tool sheets [35], and a perception sheet) and a tool package comprising the program (Table 1) were used for evaluation.

**Table 1:** Program instruments

Type of tool	When was it used?	Who used it and for whom?	Contents	Development process
Power point presentation (68 slides)	First session of the intervention	KF presented it to the participants	Overall, 15–20 min. Association of social demographic changes and foot issues, various inconvenient policies and social factors that may affect foot care, foot care regulations by the Ministry of Health, Labor and Welfare, health checkups before exercise or measurement, anatomy and physiology, and assessment and care for nails and skin. Vascular- and neurologic-related foot problem and their assessment and care. Toe and foot exercises, various types of foot-associated care, including shoes and socks, infection management, and sedentary behaviors	KF developed this presentation based on item pools and repeated discussions with an illustrator to place appropriate drawing on the slides (all drawing were made on an iPad)
Motion picture materials (10 min)	First session of the intervention	KF provided it to the participants	Selected topics included (1) foot bath, (2) cutting nails using a nail clipper, (3) scaling sole of the foot using a foot file, and (4) taping for reducing pain of ingrown nail	KF planned the contents and allocation time for each topic in the motion pictures. KF contacted a foot care specialist (IY) to be a main performer on the motion pictures and discussed the contents. Then, repeated adjustments were made to the motion pictures to create a more professional appearance
Picture story cards (19 pages)	Former part of the intervention	Participants demonstrated this in front of clients	Story: History of human beings. Human beings on the planet. Human body as a small universe Life review: Importance of foot exercises. Pictures of foot and toe exercises	KF created the story and asked an illustrator to draw 19 pages of pictures. Illustrations were drawn using an iPad
Foot care booklet (78 pages)	Former part of the intervention	Participants	Contents of this booklet were similar to those in the power point presentation, with some additional information	KF created this booklet based on item pools and repeated discussions with an illustrator to place appropriate drawing in the booklet. KF created a foot care assessment sheet in the booklet Contents of the foot care booklet were reviewed by experts
Foot care assessment sheet (1 page)	Latter part of the intervention	Participants		Based on the scoring system developed in other researcher’s study [49], KF created the foot care assessment sheet in the booklet. KF obtained approval for translation and the use of some portions of his scoring system. (KF keeps records in email exchange with the researcher). Contents of the foot care assessment sheet were reviewed by experts
Foot care advice card (1 point)	Distributed after the third or fourth session of the intervention	Participants	Majority of the content are answers to foot care knowledge and practice questionnaires	KF summarized the essence of foot care including the answers to foot care knowledge and practice questions and then condensed this information into a telephone card-sized sheet that can easily be put in a wallet. KF asked the participants to read it before answering the questionnaires after the intervention and to put it in their purse so they could always review it
Foot care kit (foot and toe nail files)	A foot file was given to a provider. A toe nail file was given to each participant	KF and participants used these for clients	The foot file was used for corns, calluses, or thickness of sole keratin	KF purchased these from a foot care salon in Tokyo, where KF acquired skills for foot care
Bamboo stepping equipment (for sole stretching)	On rent for use during the sessions	KF and participants used this for clients	When a person steps on the bamboo stepping equipment, it stimulates the overall sole, including the bones, muscle, circulation, and nerves, which achieve better body improvement	KF purchased this from a manufacturing company and used it during the intervention

All tools were developed by the first author. The features of the tools were integrated as a foot care learning package and prepared as introductory tools for participants who did not receive sufficient foot care education.

#### Data analysis

Data were analyzed with descriptive statistics. Correlations of changes in foot care knowledge and practice scores with performance scores for each tool were analyzed using Pearson’s correlation coefficient. Changes in foot care knowledge and practice scores were analyzed using a *t* test; performance scores were calculated by summing scores from descriptive statistics.

### Results

#### Primary outcomes

**Fig. 1:**
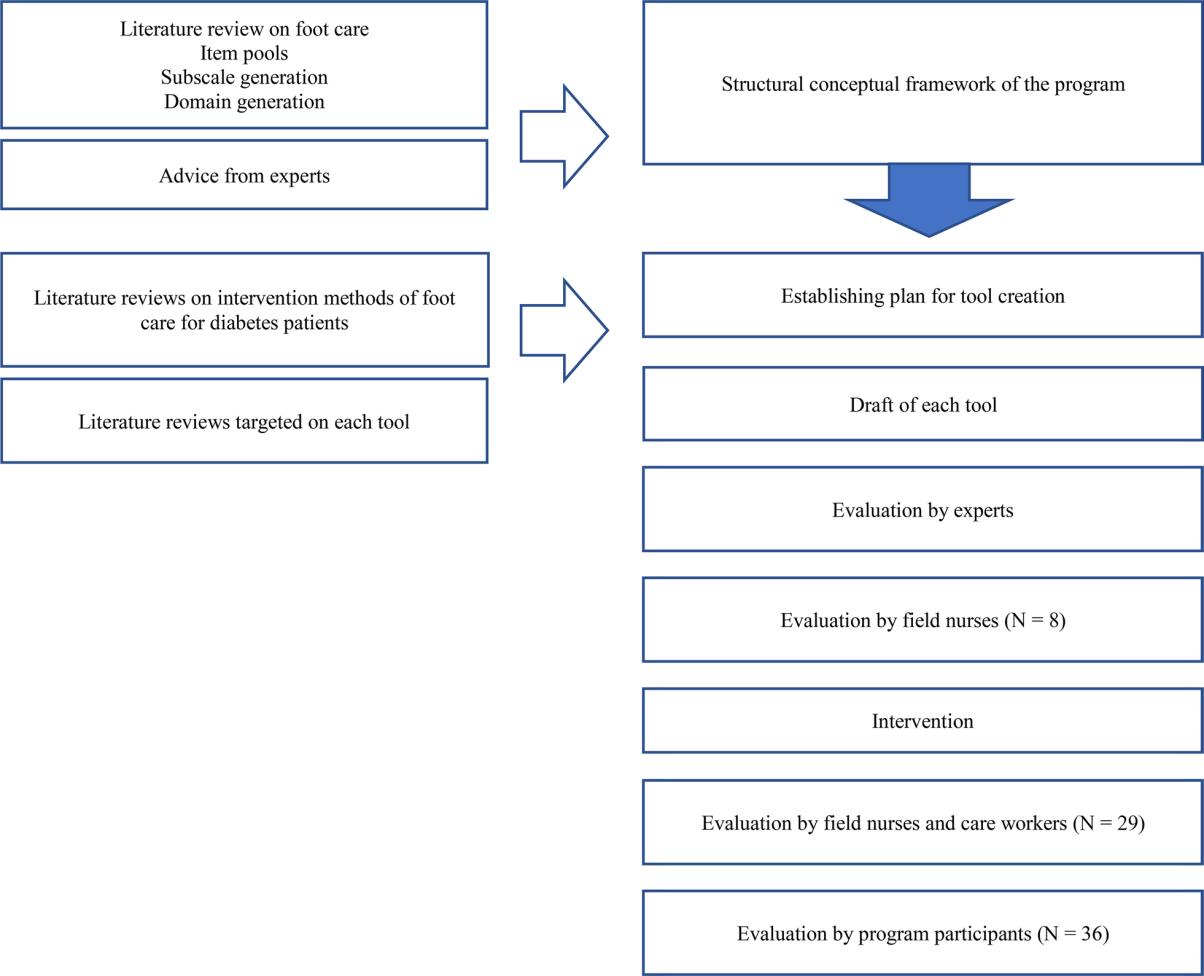
Flow chart of the program evaluation

Figure 1 shows program evaluation flow.

Performance scores correlated with skin assessment, skin practices, and consultation in the practice category subscales and the total practice score (Table 2), with no correlations between knowledge and performance scores. Skin assessment and consultation of practice subscale items and total practice scores were significantly associated with performance score (*p*-value, 0.005, 0.027, 0.017 respectively).

**Table 2:** Correlation between foot care knowledge/practice and performance scores (N = 36)

Subscale items: knowledge	Pearson	*p*-value
Nail	0.076	0.661
Skin	− 0.207	0.226
Vascular and neurological disorders	− 0.259	0.128
Toe and arch	− 0.109	0.526
Infection	0.094	0.584
Shoes and socks	− 0.076	0.661
Sedentary behavior	0.070	0.686
Total	− 0.200	0.242
Skin assessment	0.457	0.005**
Nail	0.161	0.350
Skin	0.307	0.069
Hygiene	0.198	0.248
Movement and toe exercise	0.153	0.374
Consultation	0.369	0.027*
Total	0.397	0.017*

* *p *< 0.05, ** *p *< 0.01*** *p *< 0.001

#### Secondary outcomes

The MPs and foot care booklet were evaluated. Five questions were asked regarding ease of understanding, appropriate length, gaining new knowledge, and the future usability of the MPs and PPT. Given the limited number of evaluators, qualitative comments were weighted as references based on two classifications of skill learning possibility and the structure of the contents see Additional file 1. Five participants noted that excess information was included in the foot care booklet and that the main points should be emphasized to create a smaller pamphlet. For the motion pictures, two of the participants also noted that a large amount of information was included, while other topics that were not included in the MPs were extracted as additional learning topics from the respondents.

#### Tertiary outcome

Twenty-nine participants evaluated the PPT and MPs [mean scores: 3.8–4.1 (SD 0.62–0.9), which are above average] see Additional file 2.

Three participants noted that the presenter’s speed was extremely fast and difficult to follow. Three participants stated the desire to learn about nail cutting. Some participants recommended including rehabilitation for foot and practical cases.

### Discussion

We evaluated the association between the performance scores obtained using the created tools and improvement in foot care knowledge and practices and evaluated the development process of these tools and their effectiveness.

Correlation analysis revealed that the participants’ performance scores were related to skin assessment, skin practice, and total consultation practice scores. The original tools as a package helped to provide the essence of most learning methods and pyramids proposed by the National Training Laboratories for Applied Behavioral Sciences [36].

Skin assessment and practice were emphasized in the PPT, MPs, foot care booklet, and one-point foot care card. Foot parts that tend to be overlooked, such as the heel, sole, or skin between the toes, were emphasized so that the participants could learn assessment skills for these parts. Given the high prevalence of nail and skin fungal infections [37], signs for the detection of these infections were covered in some tools. The first author (KF) described the adverse effects of daily sole washing, the risk area of the skin between the toes, and the importance of applying ointments after wiping or washing the skin to avoid stacking ointment. Foot hygiene was covered in the program and has been reported previously [38, 39]; however, it was not included in the practice session due to the providers’ work circumstances.

Time spent on foot care practices should be considered by in-home service providers using hands-on practical materials with appropriate training personnel. Points of care stated in other scales (nail, hygiene, and sedentary) were also explained; however, additional hands-on practice may be necessary. Nail practice is particularly difficult because of the complexity of nail problems with aging. Studies limited to specific areas in Japan have reported an association between nail problems such as ingrown nails and reduced limb function [40, 41]. Nurses and care workers may encounter individuals with various nail problems; however, limited resources or a lack of access to foot care professionals may cause uncertainty among NCWs regarding their capability to provide foot care. Therefore, appropriate nail care methods and materials for in-home NCWs should be developed. There was no significant association between changes in knowledge and performance scores, indicating that time constraints affect the ability to learn various topics in a short time.

Community-dwelling older people with foot problems must seek help [5, 42]. Tinea pedis and tinea unguium are among the top five skin disorders in individuals aged ≥ 60 years [43]. Predicting foot ulcers via early screening prevents further worsening of the condition [7, 44]. Early problem detection and consultation with other professionals for referral judgment by NCWs are important for foot and general health. The contents of the tools include information on when and what type of foot problems require doctor referrals, highlighting urgent (e.g., sudden coldness of one foot) and recommended (fungal infection on nails or skin) examples.

Overall, the described program is important as an introductory intervention for NCWs caring for older people. As successful educational foot care programs for patients or clients [45], multifaceted interventions modify emotions and perceptions [46], leading to changes in behavior and care practices and fall prevention [47]. Although we observed an association between the overall practice and performance scores, each tool should be evaluated in detail in the future. Additional tools should be considered in the future to adjust for differences in caregivers’ educational backgrounds.

## Limitations

In contrast to podiatrists in other countries [48], there is no guarantee that referred patients will see a doctor who has adequate foot care knowledge. Such patients may feel disappointed or may be referred to a different doctor. Furthermore, despite the presence of some foot care-related certificates from different associations, foot care professionals are limited.

Foot care programs may include excess information that must be applied in a short period, and NCWs may require additional practical and hands-on information. Acquiring proper nail care skills requires time, extensive learning, and experience. Basic treatment skills, such as using nail files or treatment for reducing pain due to ingrown nails, should be emphasized.

## Supplementary information

**Additional file 1.** Qualitative comments by the eight evaluators.

**Additional file 2.** Quantitative and qualitative comments by the 29 evaluators.

## Data Availability

All data generated or analyzed during this study are included in this published article and its supplementary information files. Availability of the data and materials the dataset used and analyzed during this study are available from the corresponding author.

## References

[1] World Bank Group. In: Population ages 65 and above (% of total population). 2018. https://data.worldbank.org/indicator/SP.POP.65UP.TO.ZS?end=2018&start=1960&view=chart. Accessed 25 Aug 2019.

[2] Establishing ‘the Community-based Integrated Care System. 2016 https://www.mhlw.go.jp/english/policy/care-welfare/care…/establish_e.pdf. Accessed 23, June 2020.

[3] Fujii K (2019). Effect of foot care interventions for older adults using day care services. Nurs Open..

[4] Mitty E (2009). Nursing care of the aging foot. Geriatr Nurs..

[5] Miikkola M, Lantta T, Suhonen R, Stolt M (2019). Challenges of foot self-care in older people: a qualitative focus-group study. J Foot Ankle Res..

[6] Stolt M, Suhonen R, Puukka P, Viitanen M, Voutilainen P, Leino-Kilpi H (2013). Nurses’ foot care activities in home health care. Geriatr Nurs..

[7] Campbell J (2006). Characteristics of the foot health of ‘low risk’ older people: a principal components analysis of foot health measures. Foot..

[8] Menz HB (2016). Chronic foot pain in older people. Maturitas..

[9] Guidozzi F (2017). Foot problems in older women. Climacteric..

[10] Stolt M, Suhonen R, Puukka P, Viitanen M, Voutilainen P, Leino-Kilpi H (2013). Development process and psychometric testing of foot health assessment instrument. J Clin Nurs.

[11] Kaya Z, Karaca A (2018). Evaluation of nurses’ knowledge levels of diabetic foot care management. Nurs Res Pract..

[12] Uritani D, Fukumoto T, Matsumoto D, Shima M (2015). Associations between toe grip strength and hallux valgus, toe curl ability, and foot arch height in Japanese adults aged 20 to 79 years: a cross-sectional study. J Foot Ankle Res..

[13] Suwa M, Imoto T, Kida A, Yokochi T, Iwase M, Kozawa K (2018). Poor toe flexor strength, but not handgrip strength, is associated with the prevalence of diabetes mellitus in middle-aged males. Endocr J.

[14] Keevil VL, Wijndaele K, Luben R, Sayer AA, Wareham NJ, Khaw KT (2015). Television viewing, walking speed, and grip strength in a prospective cohort study. Med Sci Sports Exerc.

[15] Mickle KJ, Caputi P, Potter JM, Steele JR (2016). Efficacy of a progressive resistance exercise program to increase toe flexor strength in older people. Clin Biomech.

[16] Ernst E (2003). The safety of massage therapy. Rheumatology.

[17] Hampton S (2010). Chronic oedema and lymphoedema of the lower limb. Br J Community Nurs..

[18] Sharoni SK, Rahman HA, Minhat HS, Shariff-Ghazali S, Ong MHA (2018). The effects of self-efficacy enhancing program on foot self-care behaviour of older adults with diabetes: a randomised controlled trial in elderly care facility, Peninsular Malaysia. PLoS ONE..

[19] Keller-Senn A, Probst S, Imhof RM, Imhof L (2015). Nurse-led education programme enhancing foot care self-efficacy in high-risk diabetes population: pilot randomised controlled study. Int Diabetes Nurs..

[20] Mackie S (2006). Developing an education package on diabetic foot disease. Br J Community Nurs..

[21] Fan L, Sidani S, Cooper-Brathwaite A, Metcalfe K (2013). Feasibility, acceptability and effects of a foot self-care educational intervention on minor foot problems in adult patients with diabetes at low risk for foot ulceration: a pilot study. Can J Diabetes..

[22] Nguyen TPL, Edwards H, Do TND, Finlayson K (2019). Effectiveness of a theory-based foot care education program (3STEPFUN) in improving foot self-care behaviours and foot risk factors for ulceration in people with type 2 diabetes. Diabetes Res Clin Pract.

[23] Hassan ZM (2017). Mobile phone text messaging to improve knowledge and practice of diabetic foot care in a developing country: feasibility and outcomes. Int J Nurs Pract..

[24] Kolltveit BC, Gjengedal E, Graue M, Iversen MM, Thorne S, Kirkevold M (2016). Telemedicine in diabetes foot care delivery: health care professionals’ experience. BMC Health Serv Res..

[25] Tchero H, Noubou L, Becsangele B, Mukisi-Mukaza M, Retali GR, Rusch E (2017). Telemedicine in diabetic foot care: a systematic literature review of interventions and meta-analysis of controlled trials. Int J Low Extrem Wounds..

[26] Nesari M, Zakerimoghadam M, Rajab A, Bassampour S, Faghihzadeh S (2010). Effect of telephone follow-up on adherence to a diabetes therapeutic regimen. Jpn J Nurs Sci..

[27] Kasmaienezhadfard S, Pourrajab M, Rabbani M (2015). Effects of pictures in textbooks on students’ creativity. Multi Discip Edu Global Quest, br..

[28] Brame CJ (2016). Effective educational videos: Principles and guidelines for maximizing student learning from video content. CBE Life Sci Educ..

[29] Toppin IN (2011). Video lecture capture (VLC) system: a comparison of student versus faculty perceptions. Educ Inf Technol..

[30] Kinnari-Korpela H (2015). Using short video lectures to enhance mathematics learning-experiences on differential and integral calculus course for engineering students. Inform Educ Int J..

[31] Giannakos MN, Jaccheri L, Krogstie J (2016). Exploring the relationship between video lecture usage patterns and students’ attitudes. Br J Educ Technol..

[32] Clements J. Storytelling flip over picture book and method of providing and presenting a story. Google Patents. 1998.

[33] Shekelle PG, Woolf SH, Eccles M, Grimshaw J (1999). Developing guidelines. BMJ.

[34] Ministry of Health, Labour and Welfare. In: Long-term care insurance system of Japan. 2016. https://www.mhlw.go.jp/english/policy/care-welfare/care-welfare-elderly/dl/ltcisj_e.pdf. Accessed 20 Jan 2020.

[35] Fujii K, Stolt M (2020). Intervention study of a foot-care programme enhancing knowledge and practice among nurses and care workers at in-home service providers. Nurs Open..

[36] Wood EJ (2004). Problem-based learning: exploiting knowledge of how people learn to promote effective learning. Biosci Educ..

[37] Suzuki S, Mano Y, Furuya N, Fujitani K (2017). Epidemiological Study on Trichophyton disseminating from the feet of the elderly. Jpn J Hygine..

[38] Voegeli D (2008). The effect of washing and drying practices on skin barrier function. J Wound Ostomy Continence Nurs..

[39] Martini J, Huertas C, Turlier V, Saint-Martory C, Delarue A (2017). Efficacy of an emollient cream in the treatment of xerosis in diabetic foot: a double-blind, randomized, vehicle-controlled clinical trial. J Eur Acad Dermatol Venereol.

[40] Imai A, Takayama K, Satoh T, Katoh T, Yokozeki H (2011). Ingrown nails and pachyonychia of the great toes impair lower limb functions: improvement of limb dysfunction by medical foot care. Int J Dermatol.

[41] Yamashita T, Yamashita K, Rinoie C, Takase Y, Sato M, Yamada K (2019). Improvements in lower-limb muscle strength and foot pressure distribution with foot care in frail elderly adults: a randomized controlled trial from Japan. BMC Geriatr..

[42] Stolt M, Suhonen R, Puukka P, Viitanen M, Voutilainen P, Leino-Kilpi H (2012). Foot health and self-care activities of older people in home care. J Clin Nurs.

[43] Furue M, Yamazaki S, Jimbow K, Tsuchida T, Amagai M, Tanaka T (2011). Prevalence of dermatological disorders in Japan: a nationwide, cross-sectional, seasonal, multicenter, hospital-based study. J Dermatol.

[44] Boyko EJ, Ahroni JH, Cohen V, Nelson KM, Heagerty PJ (2006). Prediction of diabetic foot ulcer occurrence using commonly available clinical information: the Seattle Diabetic Foot Study. Diabetes Care.

[45] Wylie G, Torrens C, Campbell P, Frost H, Gordon AL, Menz HB (2019). Podiatry interventions to prevent falls in older people: a systematic review and meta-analysis. Age Ageing.

[46] Scollan-Koliopoulos M, Walker EA, Bleich D (2010). Perceived risk of amputation, emotions, and foot self-care among adults with type 2 diabetes. Diabetes Educ..

[47] Spink MJ, Menz HB, Fotoohabadi MR, Wee E, Landorf KB, Hill KD (2011). Effectiveness of a multifaceted podiatry intervention to prevent falls in community dwelling older people with disabling foot pain: randomised controlled trial. BMJ.

[48] Wylie G, Menz HB, McFarlane S, Ogston S, Sullivan F, Williams B (2017). Podiatry intervention versus usual care to prevent falls in care homes: pilot randomised controlled trial (the PIRFECT study). BMC Geriatr..

[49] Menz HB, Lord SR (2001). The contribution of foot problems to mobility impairment and falls in community-dwelling older people. J Am Geriatr Soc.

