# Supplementary material for: Evaluation of the development process and effects of a foot care program with educational tools for nurses and care workers as in-home service providers
Source: BMC Res Notes. 2020 Sep 5;13:418. doi: 10.1186/s13104-020-05263-3 (PMC7487692; doi:10.1186/s13104-020-05263-3)
Supplement: Supplementary file 1 — Additional file 1. Qualitative comments by the eight evaluators. [file 13104_2020_5263_MOESM1_ESM.docx]

**Additional file 1:** Qualitative comments by the eight evaluators

| Tool | Skill | Structure |
| --- | --- | --- |
| Motion pictures | It was the first time I learned how to use nail and foot files (1).  I learned how to wash the foot correctly before performing foot care (1).  I would like to learn how to cut nails with a regular nail clipper (2).  I wanted to learn more about nails and skin with fungal infection (1).  I wanted to watch NG points of foot care.  I wanted to watch how to use cotton packing for an ingrown nail (1). | Much information was included; therefore, it is difficult to retain all the information at one time (2).  Motion pictures were more useful than the foot care note (1).  It was difficult to watch the introduction part (1).  Scripts should be provided for the motion pictures (1).  It might be difficult to understand proper care of ingrown nails (1). |
| Foot care booklet |  | Much information was included, so only the important points should be included for use in the work field (5). It might be better to use a small pamphlet or picture flip card (1).  It might be difficult to retain all the knowledge and information (3).  Some pictures and explanation did not match (3).  The title should have larger font (1).  We must consider how and when we can perform foot care in the field (1). |
