# Supplementary material for: Evaluation of the development process and effects of a foot care program with educational tools for nurses and care workers as in-home service providers
Source: BMC Res Notes. 2020 Sep 5;13:418. doi: 10.1186/s13104-020-05263-3 (PMC7487692; doi:10.1186/s13104-020-05263-3)
Supplement: Supplementary file 2 — Additional file 2. Quantitative and qualitative comments by the 29 evaluators. [file 13104_2020_5263_MOESM2_ESM.docx]

**Additional file 2:** Quantitative and qualitative comments by the 29 evaluators

| Questions | | Mean | SD |
| --- | --- | --- | --- |
| MP: It was easy to understand | | 4.1 | 0.66 |
| MP: Length was appropriate | | 3.8 | 0.91 |
| MP: I could learn new knowledge from the motion pictures | | 4.1 | 0.72 |
| MP: I can use it in the field hereafter | | 3.9 | 0.65 |
| PPT: It was easy to understand | | 3.9 | 0.62 |
| PPT: The length was appropriate | | 3.9 | 0.79 |
| PPT: I could acquire knowledge from the PPT | | 4.0 | 0.78 |
| Answers: | Comments on both tools (motion pictures and power point presentation):  Knowledge and skills: I wanted to learn more about how to cut toenails with problems, such as thicker toenails, ingrown nails,etc for practical use in the field (3).  I wanted to learn more about practical skills hereafter (1).  I learned the association between foot problems and the whole body, including clench (2).  It might be useful to include the exact or prospective effects of foot care on frail older people (1).  I wanted learn more about foot rehabilitation (1).  Structure: The speed of the talk was extremely fast and difficult to follow (3).  More practical cases should be presented (1).  The entire flow of presentation and motion pictures were fine, but more in-depth information might be better for each part (1).  I learned how to cut nails and assess skin (1).  My interest in foot care has developed (1).  I would like to learn more about foot care (1). | | |

MP: Motion pictures, PPT: Power point presentation
